# Supplementary material for: 19.31% binary organic solar cell and low non-radiative recombination enabled by non-monotonic intermediate state transition
Source: Nat Commun. 2023 Mar 30;14:1760. doi: 10.1038/s41467-023-37526-5 (PMC10063688; doi:10.1038/s41467-023-37526-5)
Supplement: Supplementary file 1 — Supplementary Information [file 41467_2023_37526_MOESM1_ESM.pdf]

Supplementary Information

19.31% binary organic solar cell and low non-radiative recombination enabled by non-monotonic intermediate state transition

Jiehao Fu[1,2], Patrick W. K. Fong[1,2], Heng Liu[3], Chieh-Szu Huang[4], Xinhui Lu[3], Shirong Lu[5], Maged Abdelsamie[6,7], Tim Kodalle[8], Carolin M. Sutter-Fella[8], Yang Yang[4*] and Gang Li[1,2,*]

[1] Department of Electronic and Information Engineering, Research Institute for Smart Energy (RISE), Guangdong-Hong Kong-Macao Joint Laboratory for Photonic-Thermal-Electrical Energy Materials and Devices, The Hong Kong Polytechnic University, Hung Hom, Kowloon, Hong Kong 999077, China.

[2] The Hong Kong Polytechnic University Shenzhen Research Institute, Shenzhen 518057, China.

[3] Department of Physics, The Chinese University of Hong Kong, Shatin, Hong Kong 999077, China.

[4] Department of Materials Science and Engineering, University of California Los Angeles (UCLA), Los Angeles, CA90095, USA

[5] School of Materials Science and Engineering, Taizhou University, Taizhou 318000, P. R. China

[6] Materials Sciences Division, Lawrence Berkeley National Laboratory, Berkeley, CA 94720, USA

[7] Materials Science and Engineering Department, King Fahd University of Petroleum and Minerals, Dhahran, Saudi Arabia

[8] Molecular Foundry, Lawrence Berkeley National Laboratory, Berkeley, CA 94720, USA

*E-mail: G. L. (e-mail: gang.w.li@polyu.edu.hk); Y. Y. (email: yangy@ucla.edu)

Supplementary Figures

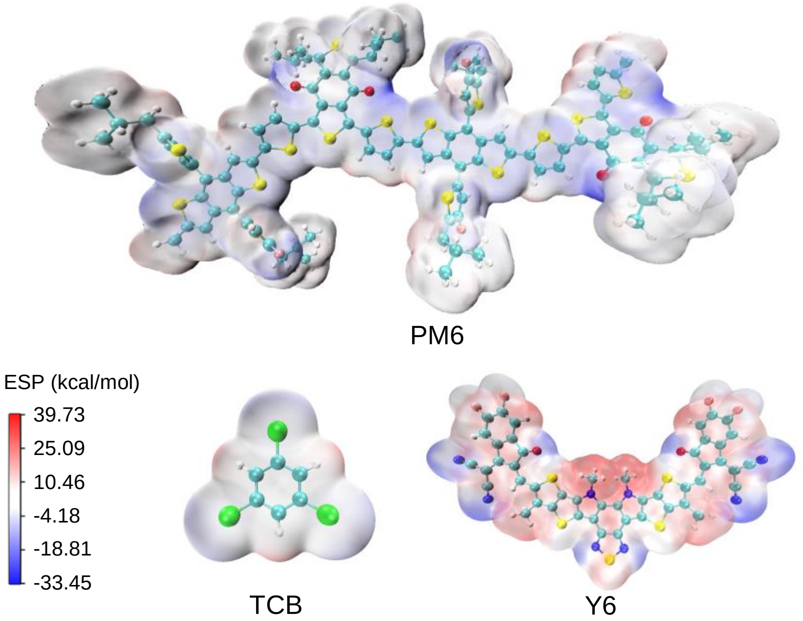

Supplementary Fig. 1 The electrostatic potential (ESP) surfaces of TCB and active materials calculated from the density functional theory (DFT) simulation at the BLYP/6–31G* level. According to the computational result, in TCB molecule, the hydrogen (H) atoms show the maximum positive ESP value. While oxygen (O) atoms in carbonyl groups (-CO) and nitrogen (N) atoms in cyano groups (-CN) show the minimum negative ESP value in PM6 molecule and Y6 molecule, respectively. This indicates the interaction between TCB and light absorbing materials originates from hydrogen bonds, i.e. -CO…H- and -CN…H-.

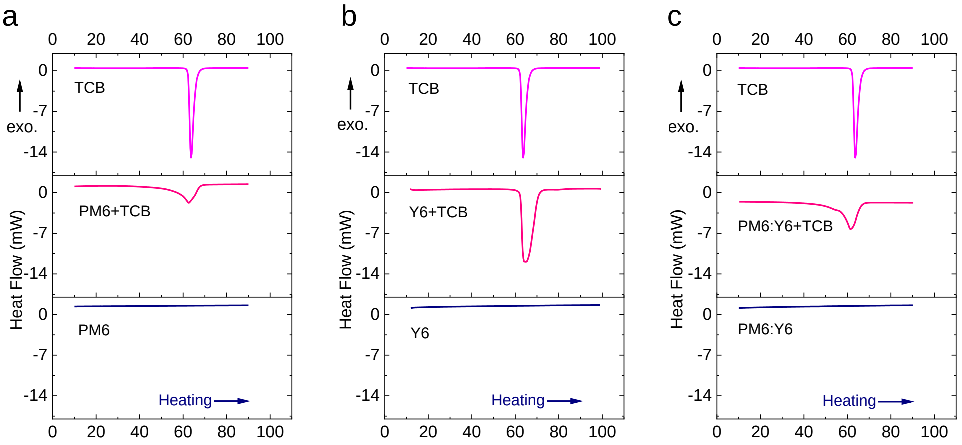

Supplementary Fig. 2 Thermal behaviors between TCB and active materials. **a** DSC thermograms (heating process) of PM6, PM6:TCB and TCB. **b** DSC thermograms (heating process) of Y6, Y6:TCB and TCB. **c** DSC thermograms (heating process) of PM6:Y6, PM6:Y6:TCB and TCB.

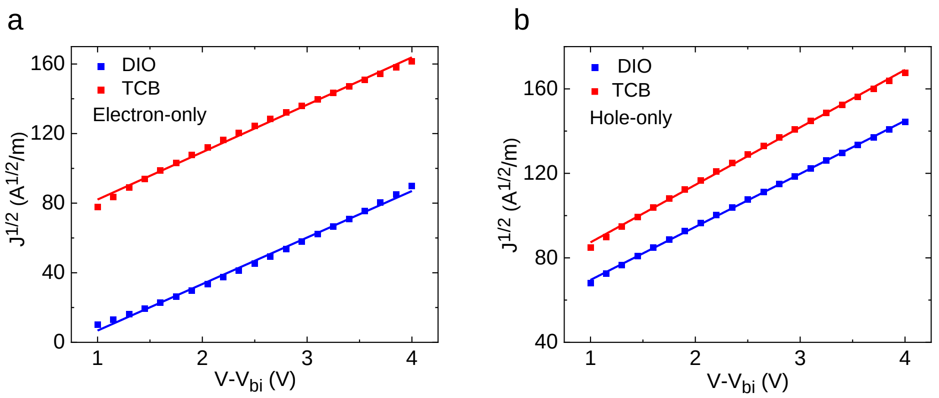

Supplementary Fig. 3 SCLC measurements. The dark $J^{1/2}$-V curves of electron-only (**a**) and hole-only (**b**) PM6:Y6-based devices with different treatments.

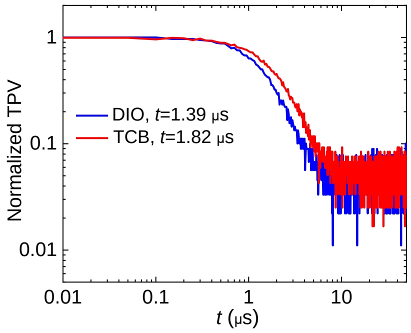

Supplementary Fig. 4 TPV measurements of PM6:Y6-based OSCs with different treatments.

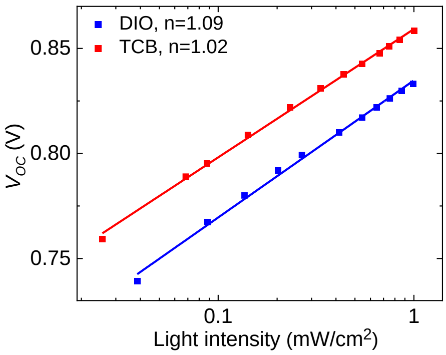

Supplementary Fig. 5 V_{OC} versus light intensity of PM6:Y6-based OSCs with different treatments.

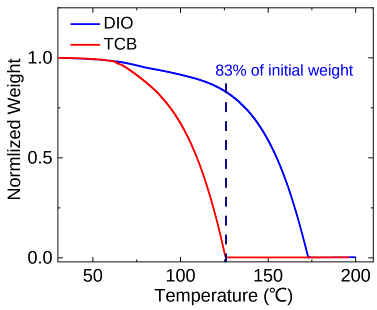

Supplementary Fig. 6 Thermogravimetric analysis (TGA) measurements of DIO and TCB with a heating rate of 1℃/min. Finally, 83% of DIO remains in crucible when TCB is totally removed, demonstrating the excellent volatility of TCB even though it is solid at room temperature.

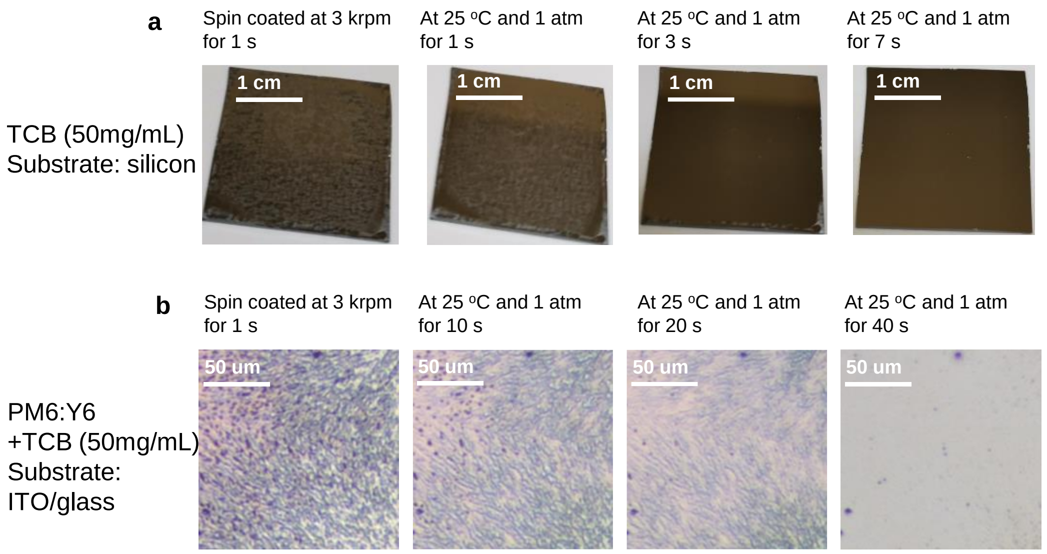

Supplementary Fig. 7 The excellent volatility of TCB. **a** Pure TCB (50mg/mL in CF) was spin coated on silicon at 3 krpm for 1s, then kept in ambient air condition (25℃ and 1 atm): just after 7s, TCB cannot be observed visually. These pictures were captured with a digital camera (on the same sample at different time). **b** PM6:Y6 (16mg/mL in CF, D:A=1:1.2) with TCB (50mg/mL) was spin coated on ITO substrate at 3 krpm for 1s, then kept under microscope for in situ observation in ambient air condition (25℃ and 1 atm): after 40s, TCB cannot be observed at micrometer scale. These pictures were taken with a microscope CCD camera.

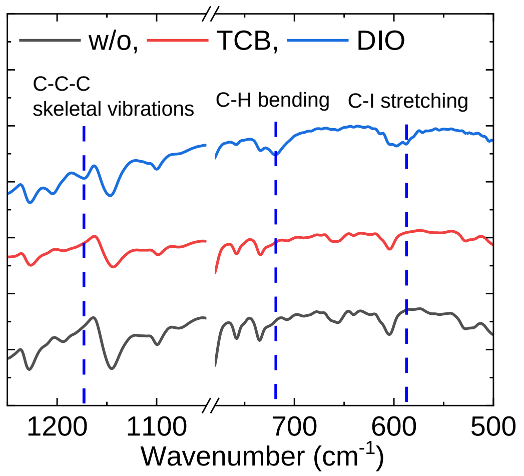

Supplementary Fig. 8 FTIR spectra of PM:Y6 films with different treatments after being spin coted at 3 krpm for 30 s. The TCB treated film shows the same FTIR spectra as the film without additive, but the DIO treated film shows several different peaks attributing to the residue of DIO (blue dashed line positions).

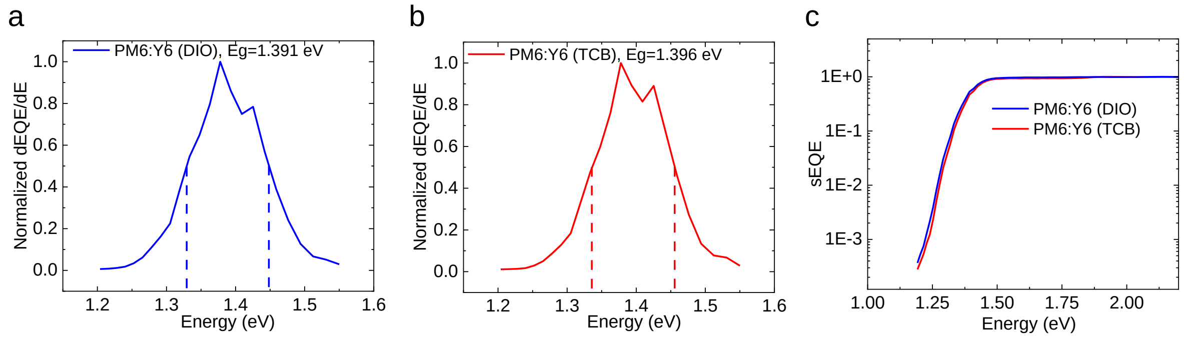

Supplementary Fig. 9 Determination of the E_g of the DIO processed (**a**) and TCB processed (**b**) PM6:Y6 devices via the derivatives of the EQE spectra. **c** Highly sensitive EQE curves for PM6:Y6 devices made with different treatments.

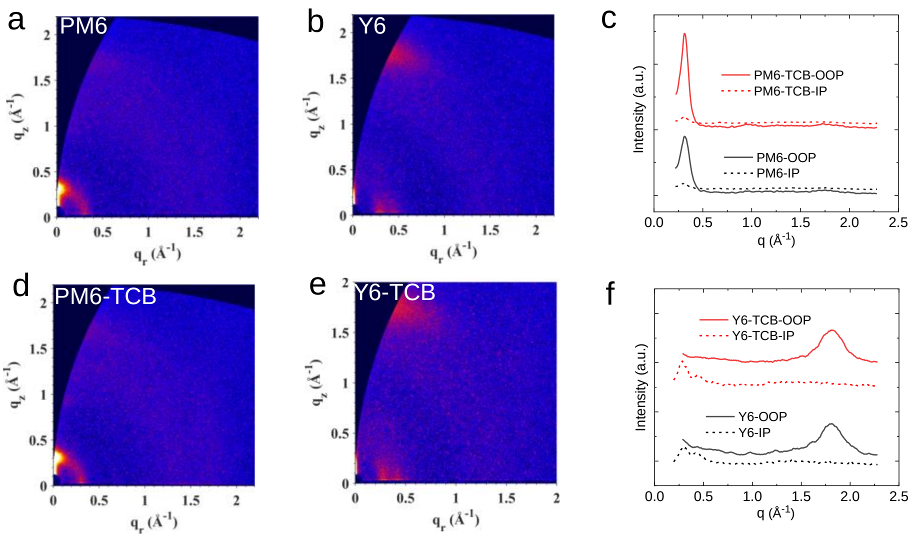

Supplementary Fig. 10 The impact of TCB on molecular stacking. 2D GIWAXS diffraction patterns of neat PM6 (**a**, **b**) and neat Y6 films (**d**, **e**) with and without TCB treatment. 1D GIWAXS diffraction patterns of neat PM6 (**c**) and neat Y6 (**f**) films with and without TCB treatment.

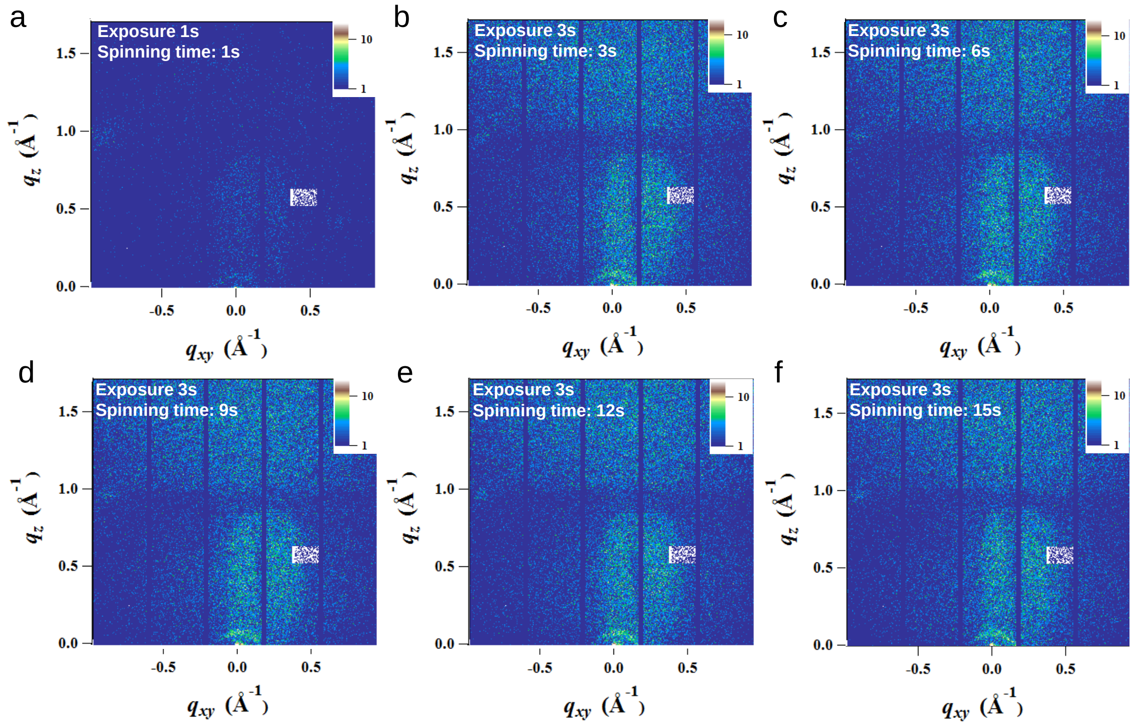

Supplementary Fig. 11 Time-resolved GIWAXS patterns of TCB treated samples during spin coating process. a Spinning time of 1s with exposure time of 1s. Spinning time of 3s (**b**), 6s (**c**), 9s (**d**), 12s (**e**), and 15s (**f**) with exposure time of 3s.

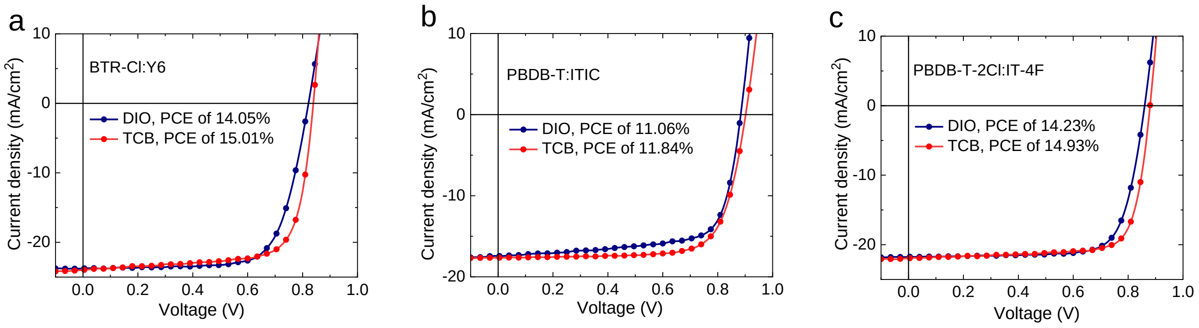

Supplementary Fig. 12 *J*–*V* curves for the DIO processed and TCB processed OSCs based on BTR-Cl:Y6 (**a**), PBDB-T:ITIC (**b**), PBDB-T-2Cl:IT-4F (**c**).

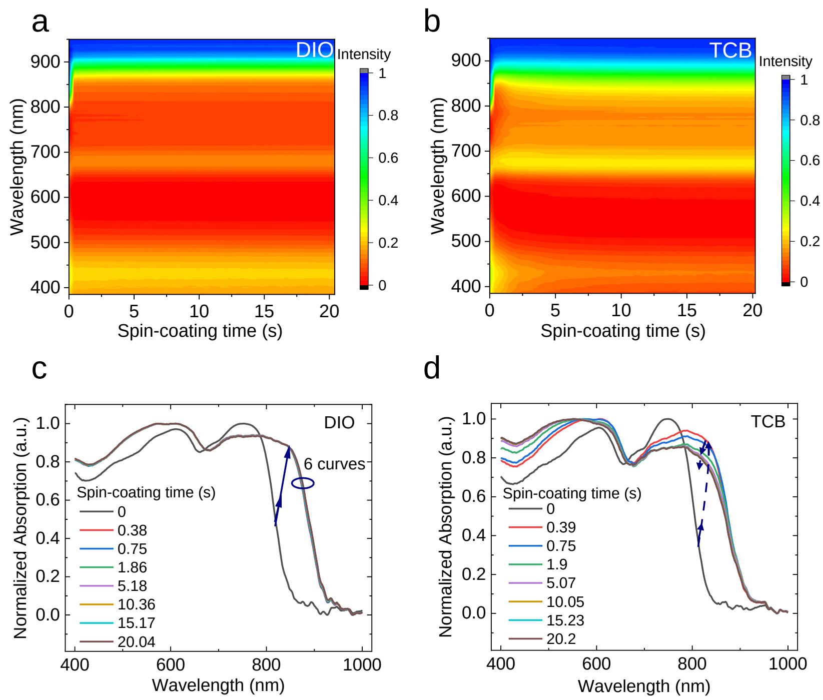

Supplementary Fig. 13 In situ UV-vis characterization. The color mapping of in situ UV-vis reflectance spectra as a function of spin-coating time for PM1:BPT-eC9 blends with DIO (**a**) and with TCB (**b**). Normalized absorption spectra (here we defined the absorption of sample as the difference between the reflectance of background and the reflectance of sample) at representative time points for PM1:BPT-eC9 blends with DIO (**c**) and with TCB (**d**).

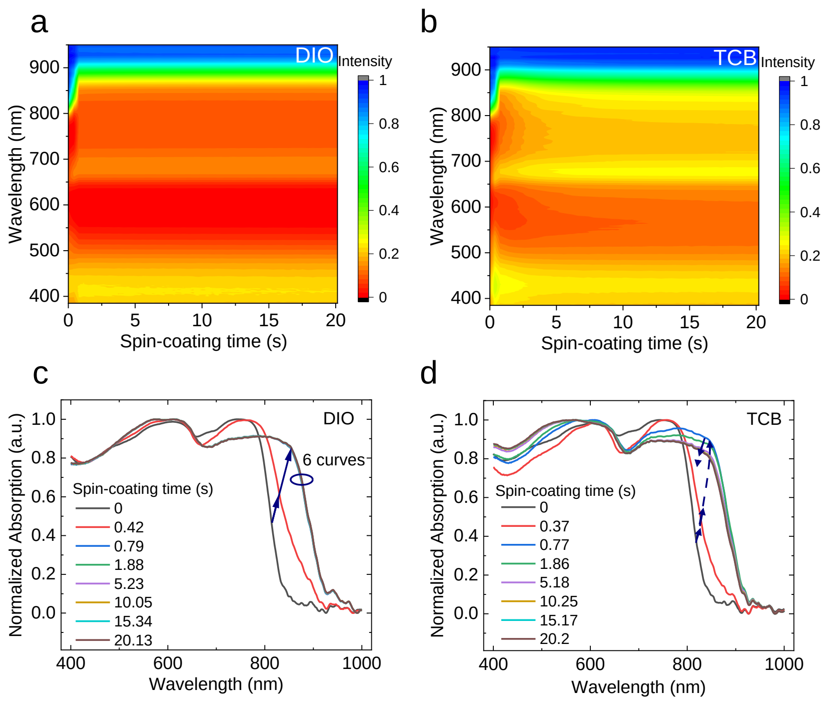

Supplementary Fig. 14 In situ UV-vis characterization. The color mapping of in situ UV-vis reflectance spectra as a function of spin-coating time for PM6:BPT-eC9 blends with DIO (**a**) and with TCB (**b**). Normalized absorption spectra (here we defined the absorption of sample as the difference between the reflectance of background and the reflectance of sample) at representative time points for PM6:BPT-eC9 blends with DIO (**c**) and with TCB (**d**).

測試報告

Report of Test

儀 器 名 稱<br>Device Name	Solar Cell
廠 牌 型 號<br>Model No.	Organic Photovoltaic Solar Cells
儀 器 序 號<br>Serial No.	12-38
測 試 日 期<br>Test Date	2022 / 05 / 27
送 測 單 位<br>Applicant	Department of Electronic and Information Engineering, Research Institute for Smart Energy (RISE), The Hong Kong Polytechnic University
送測單位地址<br>Applicant Address	The Hong Kong Polytechnic University, Hung Hom, Kowloon, Hong Kong

上項儀器經本實驗室量測，結果如內文。本報告含封面及內文共 6 頁，分離使用無效。

The test device is measured by the laboratory and the results are given in the content. The report consists of 6 pages including the cover and is invalid if separated.

報告簽署人/Approved by：

報告簽署暨發行日期/Date：2022-6-23

實驗室地址(Laboratory Address)：
高雄市路竹區路科五路 96 號 1 樓 A 區 (A area, 1F., No.96, Luke 5th Rd., Kaohsiung, Taiwan, R.O.C)

測試報告使用說明 / Report Instruction

1. 光焱科技光電校正實驗室執行測試所產生之測試結果詳列於本報告內。

   本報告之測試結果僅對報告內提及之送測件有效。

   The tested results of the device are given in the report.
   The tested results of this report are responsible to the tested device.

2. 除特別聲明外，報告內數值係在本實驗室環境下執行測試所得的結果。

   爾後使用該送測件時，其準確度與精密度將因使用時之環境狀況與使用頻率等因素而有所不同。

   Numeric data in this report are the tested results in the ambient condition of the laboratory except special statements.
   Precision and accuracy of the test device will depend on the ambient condition and usage frequency of it afterward.

3. 本報告之結果業經本實驗室之主管審核確認。

   The calibration report should be approved by head of the calibration laboratory.

4. 本報告未得到本實驗室書面同意，不得任意摘錄複製使用，但全文複製除外。

   The calibration report should not be reproduced expect in full.

(以下空白/Blank)

TEL : +886-7-6955669 FAX : +886-7-6955785

測試環境條件 Environment condition

模擬器種類 Simulator type	Steady-State Class A
照度 Irradiance	1000 W/m^2
待測件溫度 DUT Temperature	24.9 °C
環境溫度 Environmental Temperature	26.0 °C

測試使用之標準件 Standard for Calibration

儀器名稱 Device Name	Reference Cell		
廠牌型號 Model No.	SRC2020		
儀器序號 Serial No.	SRC-00170		
校正機構 Cal. Laboratory	NREL		
報告編號 Report No.	2037		
追溯日期 Cal. Date	2021 / 05 / 06		

標準件之溯源 Standard Traceability

儀器名稱 Device Name	Primary Reference cell		
廠牌型號 Model No.			
儀器序號 Serial No.	S09		
校正機構 Cal. Laboratory	NIST		
報告編號 Report No.			
追溯日期 Cal. Date	2020 / 04 / 07		
有效日期 Due Date			

TEL : +886-7-6955669 FAX : +886-7-6955785

测試結果 Test Results

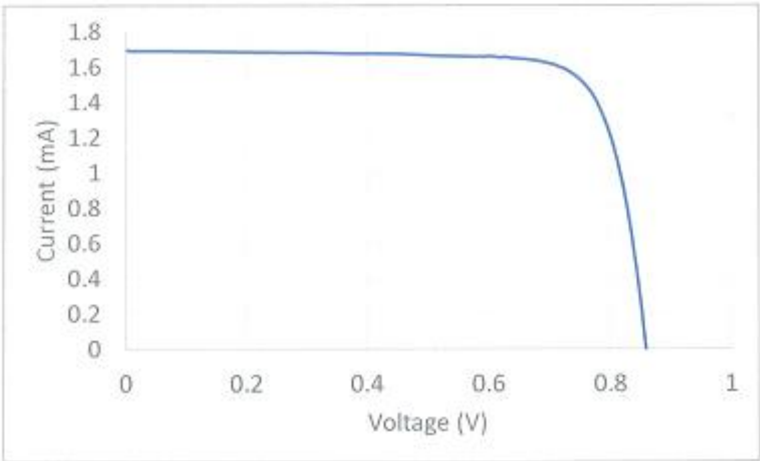

V_{OC}	=	858.56	mV	±	3.43	mV
I_{SC}	=	1693.77	μA	±	24.05	μA
P_{MPP}	=	1151.20	μW	±	16.92	μW
V_{MPP}	=	735.00	mV	±	2.94	mV
I_{MPP}	=	1566.26	μA	±	22.24	μA
FF	=	79.16		±	0.24	%
Efficiency	=	18.93		±	0.29	%
Aperture area	=	6.08	mm^2			

Contacting method: 4-wire connection
Sweep voltage range: -0.2 V ~ 1.2 V
Sweep rate: 0.13 s
Sweep direction: Isc --> Voc.

TEL : +886-7-6955669 FAX : +886-7-6955785

測試說明 Description of the test object

1 測試日期 Test Date：

1.1 測試件接收日期 Date of Receipt：2022 / 05 / 26

1.2 測試件校正日期 Date of Test：2022 / 05 / 27

2 測試地點 Test Site：

2.1 光焱科技光電校正實驗室 Enli Tech. Optoelectronic Calibration Lab.

3 測試方法 Test Method：

3.1 待測件的測試是依據本實驗室「太陽能電池最大功率測試作業指導書」[1]執行，於「IEC 60904-1 Photovoltaic devices - Part 1: Measurement of photovoltaic current-voltage characteristics」所定義之標準測試條件(STC)下進行測試，使用「IEC 60904-9 Photovoltaic devices - Part 9: Classification of solar simulator characteristics」所定義之 3A 等級穩態太陽光模擬器進行輻照，並根據「IEC 60904-7 Photovoltaic devices - Part 7: Computation of the spectral mismatch correction for measurements of photovoltaic devices」來計算光譜失配係數。太陽光譜模擬器的光譜使用光譜儀測量，並依據「IEC 60904-8 Photovoltaic devices - Part 8: Measurement of spectral responsivity of a photovoltaic (PV) device」使用單光儀測量待測件的光譜響應（或量子效率）。

The testing of the DUT was performed at Standard Testing Conditions (STC) in accordance with IEC 60904-1 Photovoltaic devices - Part 1: Measurement of photovoltaic current-voltage characteristics and Test standard operation procedure of maximum power measurement of solar cells [1] under the irradiation with a steady-state class AAA solar simulator according to IEC 60904-9 Photovoltaic devices - Part 9: Classification of solar simulator characteristics. The spectral mismatch is calculated according to IEC 60904-7 Photovoltaic devices - Part 7: Computation of the spectral mismatch correction for measurements of photovoltaic devices. The spectrum of the solar simulator is measured with a spectroradiometer. The spectral responsivity (or quantum efficiency) of the device under test is measured with a grating monochromatic according to IEC 60904-8 Photovoltaic devices - Part 8: Measurement of spectral responsivity of a photovoltaic (PV) device.

4 相對擴充不確定度 Relative Expanded Uncertainty:

4.1 相對擴充不確定度係依據本實驗室之「最大功率測試量測不確定度評估報告」[2]進行評估。

Relative expanded uncertainty is estimated based on *Estimated Uncertainty Report of maximum power measurement of solar cells [2].*

TEL : +886-7-6955669 FAX : +886-7-6955785

4.2 相對擴充不確定度係相對組合標準不確定度與涵蓋因子(*k*)之乘積，*k*=2 相對應約 95 %之信賴水準。
The relative expanded uncertainty resulting of the relative combined standard uncertainty multiplied with a coverage factor *k*=2 is specified. It corresponds to about a level of confidence of approximately 95 %.

參考文件 Reference Literature

1. LAB-3-7.2.2(1) 太陽能電池最大功率測試作業指導書
LAB-3-7.2.2(1), *Test standard operation procedure of maximum power measurement of solar cells*, Enli Technology Co., Ltd.
2. LAB-3-7.6.2(1) 最大功率測試量測不確定度評估報告
LAB-3-7.6.2(1), *Estimated Uncertainty Report of maximum power measurement of solar cells*, Enli Technology Co., Ltd.

（以下空白/Blank）

TEL : +886-7-6955669 FAX : +886-7-6955785

Supplementary Fig. 15 The copy images of PCE certificate from Enli Tech. Optoelectronic Calibration Lab. for the TCB processed PM6:BTP-eC9 OSC.

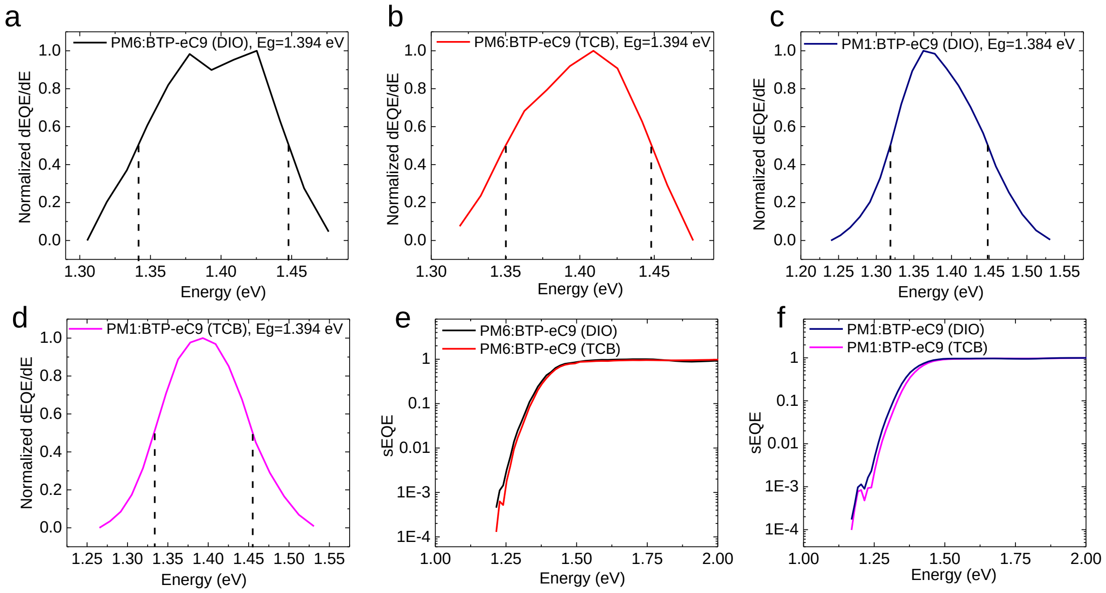

Supplementary Fig. 16 Determination of the E_g of the DIO processed PM6:BTP-eC9 device (**a**), the TCB processed PM6:BTP-eC9 device (**b**), the DIO processed PM1:BTP-eC9 device (**c**) and the TCB processed PM1:BTP-eC9 device (**d**) via the derivatives of the EQE spectra. Highly sensitive EQE curves for PM6:BTP-eC9 (**e**) and PM1:BTP-eC9 (**f**) devices made with different additives.

Supplementary Tables

Supplementary Table 1 Detailed photovoltaic parameters of three high efficiency OSC systems without additive treatments.

D:A	V_{OC} (V)	J_{SC} (mA cm^{-2})	FF (%)	PCE[a] (%)
PM6:Y6	0.858	26.15	72.01	16.16 (16.00±0.07)
PM6:BTP-eC9	0.867	26.63	70.71	16.33 (16.19±0.08)
PM1:BTP-eC9	0.903	26.52	66.23	15.86 (15.71±0.07)

[a]The average PCEs with standard deviation calculated from 20 devices in each case.

Supplementary Table 2. Photovoltaic parameters of PM6:Y6-based OSCs versus the concentration of TCB. The TCB processed device is tolerant to additive concentration, the device can maintain 17.8% efficiency (nearly 99% of the champion efficiency) within a TCB concentration range of 8-15mg/mL, which is 92-172% weight ratio compared to the donor material.

Additive	V_{OC} (V)	J_{SC} (mA cm^{-2})	FF (%)	PCE[a] (%)
w/o	0.858	26.15	72.01	16.16 (16.00±0.07)
TCB, 5mg/ml	0.856	26.59	75.56	17.20 (16.98±0.12)
TCB, 8mg/ml	0.852	26.81	78.02	17.82 (17.64±0.10)
TCB, 10mg/ml	0.852	27.02	78.43	18.06 (17.92±0.06)
TCB, 15mg/ml	0.849	27.04	77.34	17.75 (17.56±0.11)
TCB, 20mg/ml	0.840	26.71	77.43	17.37 (17.16±0.15)
TCB, 30mg/ml	0.828	26.83	76.79	17.06 (16.86±0.16)

[a] The average PCEs with standard deviation calculated from 20 devices.

Supplementary Table 3. Charge mobilities measured by SCLC method for PM6:Y6-based films with different treatments.

Condition	μ_e ($cm^2V^{-1}s^{-1}$)	μ_h ($cm^2V^{-1}s^{-1}$)	μ_e/μ_h
DIO	$3.5x10^{-4}$	$2.5x10^{-4}$	1.4
TCB	$3.6x10^{-4}$	$3.0x10^{-4}$	1.2

Supplementary Table 4. Detailed peak information from GIWAXS measurements for PM6:Y6 blend films with DIO and TCB treatments.

Q_{xy}	Location (Å^{-1})	d-spacing (Å)	Peak area
Lamellar peak-DIO	0.30	20.67	0.74
Lamellar peak-TCB	0.30	20.87	0.90
Q_z	Location (Å^{-1})	d-spacing (Å)	Peak area
π-π peak-DIO	1.82	3.45	1.35
π-π peak-TCB	1.79	3.50	1.39

Supplementary Table 5. Detailed peak information from GIWAXS measurements for neat PM6 and neat Y6 films with and without TCB treatments.

Q_{xy}	Location ($Å^{-1}$)	d-spacing (Å)	Peak area
Lamellar peak-PM6	0.30	20.87	0.20
Lamellar peak-PM6-TCB	0.31	20.53	0.19
Lamellar peak-Y6	0.30	21.30	0.34
Lamellar peak-Y6-TCB	0.29	21.97	0.47
Q_z	Location ($Å^{-1}$)	d-spacing (Å)	Peak area
Lamellar peak-PM6	0.31	20.01	1.73
Lamellar peak-PM6-TCB	0.31	20.14	2.73
π-π peak-Y6	1.81	3.46	1.31
π-π peak-Y6-TCB	1.81	3.47	1.89

Supplementary Table 6. PCEs and the corresponding non-radiative recombination loss values of recent high performance OSCs.

Active materials	EQE_{EL} (%)	ΔE_3(eV)	PCE (%)	PCE^a (%)	Reference
PM6:Y11	$3.5*10^{-2}$	0.202	16.54	16.11	1
PM6:S3:Y6	$1.9*10^{-2}$	0.223	17.53	*	2
PM6:BTP-eC9	*	0.227	17.8	17.3	3
PM6:PM7:Y6:$PC_{71}BM$	$0.94*10^{-2}$	0.240	18.07	17.35	4
PM6:L8-BO	*	0.24	18.32	17.9	5
PM6:L8-BO	$1.9*10^{-2}$	0.214	18.6	18.2	6
PB2F:PBDB-TF:BTP-eC9	$2.2*10^{-2}$	0.218	18.6	18.2	7
PM6:BTP-eC9:L8-BO-F	$3.5*10^{-2}$	0.199	18.66	18.2	8
PBDB-TF:HDO-4Cl:eC9	$5.3*10^{-2}$	0.194	18.86	18.3	9
PM1:BTP-eC9	$1.1*10^{-1}$	0.175	19.10	*	This work
PM6:BTP-eC9	$5.6*10^{-2}$	0.192	19.31	18.93	This work

[a] Certified PCE from independent third party.

Supplementary References

1 Liu, S. *et al.* High-efficiency organic solar cells with low non-radiative recombination loss and low energetic disorder. *Nat. Photon* **14**, 300-305 (2020).

2 An, Q. *et al.* Two compatible polymer donors contribute synergistically for ternary organic solar cells with 17.53% efficiency. *Energy Environ. Sci.* **13**, 5039-5047 (2020).

3 Cui, Y. *et al.* Single‐junction organic photovoltaic cells with approaching 18% efficiency. *Adv. Mater.* **32**, 1908205 (2020).

4 Zhang, M. *et al.* Single-layered organic photovoltaics with double cascading charge transport pathways: 18% efficiencies. *Nat. Commun.* **12**, 309 (2021).

5 Li, C. *et al.* Non-fullerene acceptors with branched side chains and improved molecular packing to exceed 18% efficiency in organic solar cells. *Nat. Energy* **6**, 605-613 (2021).

6 Song, J. *et al.* High-efficiency organic solar cells with low voltage loss induced by solvent additive strategy. *Matter* **4**, 2542-2552 (2021).

7 Zhang, T. *et al.* A Thiadiazole‐Based Conjugated Polymer with Ultradeep HOMO Level and Strong Electroluminescence Enables 18.6% Efficiency in Organic Solar Cell. *Adv. Energy Mater.* **11**, 2101705 (2021).

8 Cai, Y. *et al.* A Well‐Mixed Phase Formed by Two Compatible Non‐Fullerene Acceptors Enables Ternary Organic Solar Cells with Efficiency over 18.6%. *Adv. Mater.* **33**, 2101733 (2021).

9 Bi, P. *et al.* Reduced non-radiative charge recombination enables organic photovoltaic cell approaching 19% efficiency. *Joule* **5**, 2408-2419 (2021).
