# Supplementary material for: 19.31% binary organic solar cell and low non-radiative recombination enabled by non-monotonic intermediate state transition
Source: Nat Commun. 2023 Mar 30;14:1760. doi: 10.1038/s41467-023-37526-5 (PMC10063688; doi:10.1038/s41467-023-37526-5)
Supplement: Supplementary file 3 — Description of Additional Supplementary Files [file 41467_2023_37526_MOESM3_ESM.pdf]

File name: Supplementary Data 1

Description: The coordinates of the optimized computational models
