# Supplementary material for: 19.31% binary organic solar cell and low non-radiative recombination enabled by non-monotonic intermediate state transition
Source: Nat Commun. 2023 Mar 30;14:1760. doi: 10.1038/s41467-023-37526-5 (PMC10063688; doi:10.1038/s41467-023-37526-5)
Supplement: Supplementary file 5 — Reporting Summary [file 41467_2023_37526_MOESM5_ESM.pdf]

nature research

Corresponding author(s): Gang Li and Yang Yang

Solar Cells Reporting Summary

Nature Research wishes to improve the reproducibility of the work that we publish. This form is intended for publication with all accepted papers reporting the characterization of photovoltaic devices and provides structure for consistency and transparency in reporting. Some list items might not apply to an individual manuscript, but all fields must be completed for clarity.

For further information on Nature Research policies, including our data availability policy, see Authors & Referees.

Experimental design

Please check: are the following details reported in the manuscript?

1. Dimensions

Item	Reported	Details
Area of the tested solar cells	[x] Yes [ ] No	Active area of the tested solar cells is 0.11cm2
Method used to determine the device area	[x] Yes [ ] No	The active area was determined by the crossed area of counter electrode and ITO stripe.

2. Current-voltage characterization

Item	Reported	Details
Current density-voltage (J-V) plots in both forward and backward direction	[ ] Yes [x] No	Generally, organic photovoltaic devices do not have hysteresis problem. And we only scan the device in forward direction.
Voltage scan conditions<br>*For instance: scan direction, speed, dwell times*	[x] Yes [ ] No	Section "Device fabrication and testing"
Test environment<br>*For instance: characterization temperature, in air or in glove box*	[x] Yes [ ] No	In glove box at room temperature.
Protocol for preconditioning of the device before its characterization	[ ] Yes [x] No	No preconditioning protocol
Stability of the J-V characteristic<br>*Verified with time evolution of the maximum power point or with the photocurrent at maximum power point; see ref. 7 for details.*	[ ] Yes [x] No	We only tested the light stability in our lab.

3. Hysteresis or any other unusual behaviour

Item	Reported	Details
Description of the unusual behaviour observed during the characterization	[ ] Yes [x] No	No hysteresis or other unusual behaviour was observed during the characterization of the solar cells. In general, organic solar cells do not have hysteresis problems.
Related experimental data	[ ] Yes [x] No	No hysteresis or other unusual behaviour was observed during the characterization of the solar cells.

4. Efficiency

Item	Reported	Details
External quantum efficiency (EQE) or incident photons to current efficiency (IPCE)	[x] Yes [ ] No	See Fig. 2c
A comparison between the integrated response under the standard reference spectrum and the response measure under the simulator	[x] Yes [ ] No	The integrated Jsc from EQE spectrum is agreed well (less than 3% mismatch) with the Jsc from J-V measurement.
For tandem solar cells, the bias illumination and bias voltage used for each subcell	[ ] Yes [x] No	No tandem solar cell was reported in this manuscript.

5. Calibration

Item	Reported	Details
Light source and reference cell or sensor used for the characterization	[x] Yes [ ] No	Enli Technology AAA solar simulator (SS-F5) and standard Si (SRC-2020) reference cell were used during solar cells testing (Methods).
Confirmation that the reference cell was calibrated and certified	[x] Yes [ ] No	The reference cell (SRC-2020) was calibrated by NREL.

November 2017

Calculation of spectral mismatch between the reference cell and the devices under test

- [ ] Yes
- [x] No

No spectral mismatch calculation was performed in our lab.

6. Mask/aperture

Size of the mask/aperture used during testing

- [x] Yes
- [ ] No

A 0.061 cm2 aperture was used during solar cells testing (certified 0.0608 cm2, Enli Tech. Optoelectronic Calibration Lab., Taiwan, Accreditation Criteria: ISO/IEC 17025:2017).

Variation of the measured short-circuit current density with the mask/aperture area

- [ ] Yes
- [x] No

We didn't measure the solar cells with apertures of different sizes.

7. Performance certification

Identity of the independent certification laboratory that confirmed the photovoltaic performance

- [x] Yes
- [ ] No

Solar cells were certified by Enli Tech. Optoelectronic Calibration Lab., Accreditation Criteria: ISO/IEC 17025:2017

A copy of any certificate(s)
Provide in Supplementary Information

- [x] Yes
- [ ] No

See Supplementary Fig. 15

8. Statistics

Number of solar cells tested

- [x] Yes
- [ ] No

33 or 20 devices for each condition were tested.

Statistical analysis of the device performance

- [x] Yes
- [ ] No

See Table 1, Table 3, Fig. 2d, Supplementary Table 1 and Supplementary Table 2

9. Long-term stability analysis

Type of analysis, bias conditions and environmental conditions
For instance: illumination type, temperature, atmosphere humidity, encapsulation method, preconditioning temperature

- [x] Yes
- [ ] No

See Fig. 6g
